# Forecasting imbalances in the global health labor market and devising policy responses

**DOI:** 10.1186/s12960-017-0264-6

**Published:** 2018-01-11

**Authors:** Richard M. Scheffler, James Campbell, Giorgio Cometto, Akiko Maeda, Jenny Liu, Tim A. Bruckner, Daniel R. Arnold, Tim Evans

**Affiliations:** 1 School of Public Health and Goldman School of Public Policy, University of California, Berkeley, Berkeley, CA United States of America; 2 Health Workforce Department, World Health Organization, Geneva, Switzerland; 3 Organisation for Economic Co-operation and Development (OECD), Paris, France; 4 Institute for Health and Aging, Department of Social and Behavioral Sciences, University of California, San Francisco, San Francisco, CA United States of America; 5 School of Public Health, University of California, Irvine, Irvine, CA United States of America; 6 School of Public Health, University of California, Berkeley, CA United States of America; 7 The World Bank, Washington, DC, United States of America

**Keywords:** Health workforce, Global health

## Abstract

**Background:**

The High-Level Commission on Health Employment and Economic Growth released its report to the United Nations Secretary-General in September 2016. It makes important recommendations that are based on estimates of over 40 million new health sector jobs by 2030 in mostly high- and middle-income countries and a needs-based shortage of 18 million, mostly in low- and middle-income countries. This paper shows how these key findings were developed, the global policy dilemmas they raise, and relevant policy solutions.

**Methods:**

Regression analysis is used to produce estimates of health worker need, demand, and supply. Projections of health worker need, demand, and supply in 2030 are made under the assumption that historical trends continue into the future.

**Results:**

To deliver essential health services required for the universal health coverage target of the Sustainable Development Goal 3, there will be a need for almost 45 million health workers in 2013 which is projected to reach almost 53 million in 2030 (across 165 countries). This results in a needs-based shortage of almost 17 million in 2013. The demand-based results suggest a projected demand of 80 million health workers by 2030.

**Conclusions:**

Demand-based analysis shows that high- and middle-income countries will have the economic capacity to employ tens of millions additional health workers, but they could face shortages due to supply not keeping up with demand. By contrast, low-income countries will face both low demand for and supply of health workers. This means that even if countries are able to produce additional workers to meet the need threshold, they may not be able to employ and retain these workers without considerably higher economic growth, especially in the health sector.

## Introduction

Recently, there have been major reports and papers on the future shortage of health workers. One done by the World Health Organization (WHO) used a needs-based approach (see Scheffler and Cometto et al. [1]) while another done by the World Bank, and which appeared in this journal (see Liu et al. [2]), used a labor market approach. These two very different approaches produce findings that have important policy implications when the results are viewed together [3].

This paper summarizes the two approaches and the results they produce. To do this, we reran each of the models used in order to allow direct comparison of shortages and surpluses. Definitions of shortages and surpluses are consistently used in both approaches. The supply projects are the same in both approaches. But one uses a needs-based estimate and the other a demand-based estimate. The differences in these two approaches are spelled out in detail.

The results are a background for high-level policy suggestions. These broad policies would need to be refined at the country level. The results in the paper and policy suggestions provide a framework for improving health work policy around the globe.

## Background

The High-Level Commission on Health Employment and Economic Growth, co-chaired by François Hollande, President of France, and Jacob Zuma, President of South Africa, issued its report to the United Nations Secretary-General in September 2016 [4]. The report examines the creation of health and social sector jobs and identifies six causal pathways to inclusive economic growth, especially in low- and middle-income countries. Ten policy recommendations and five priority actions for the immediate 18 months following the report’s launch are presented. The underpinning rationale for the formation of the Commission and a cornerstone of its deliberations is the global mismatch between supply, need, and demand for health workers to 2030.

It has been a decade since the landmark “Working Together For Health: The World Health Report 2006” was issued by WHO [5]. This report identified a threshold on the need for health workers in the context of the Millennium Development Goals. It estimated that 2.28 skilled health professionals (midwives, nurses, and physicians) per thousand population were generally necessary to achieve 80% coverage of skilled birth attendance. This narrowly defined threshold became widely used to assess the adequacy of the supply of health workers around the globe.

As attention focused on the development of the Sustainable Development Goals (SDGs), with greater ambitions for universal access to health, addressing non-communicable diseases, mental health, and other health outcomes, the limitations of the earlier threshold became clear [6–10]. In 2013, the Global Health Workforce Alliance and WHO presented new analysis on health workforce need to 2030. Subsequently, a decision by the World Health Assembly (WHA) in 2014 led to the development and adoption of the Global Strategy on Human Resources for Health: Workforce 2030 in May 2016 by resolution WHA69.19 [11]. The Global Strategy included new analysis and estimates which quantify and project the global shortage of health workers. The analysis drew upon two reports that were developed as complementary perspectives of the global health workforce labor market in 2013 and 2030. WHO’s “Health workforce requirements for universal health coverage and the Sustainable Development Goals” quantifies through an innovative empirical approach the health workforce requirements for the attainment of SDG 3 [1]. It suggests a new benchmark of 4.45 physicians, nurses, and midwives per thousand population, identifying a substantial needs-based shortage in low-income countries. The second paper, “Global Health Workforce Labor Market Projections for 2030,” estimates and projects the demand for health workers [2]. The demand approach calculates and projects a much higher global demand-based shortage of health workers and highlights the difference between the needs-based and demand-based analysis of the global shortages of health workers.

## Methods

### Conceptual framework

To conceptualize how needs-based and demand-based shortages are calculated, we outline the labor market that exists in many countries [12–14]. We discuss three concepts in turn: need, demand, and supply for health workers. Need can be defined generally as the number of health workers required to attain the objectives of a health system. A country’s need is often estimated based on a threshold of minimum availability of health workers to address priority population health issues. The specific definition of need used in this paper is explained in the following section. Demand is the number of health workers that a health system can support in terms of funded positions or economic demand for services. Demand is correlated with the spending on health by the government, private insurance, and out-of-pocket payments. The supply of health workers refers to the number of health workers that are available in a country. Supply of health workers is a function of the training capacity in a country and the net migration, deaths, and retirements of health workers. Graphical depictions of (1) how the labor market for health professionals relates to a country’s education and health care system [15] and (2) how need, demand, and supply interrelate are available in the Appendix.

### SDG-based health worker need

The WHO report develops a novel approach that uses SDG 3 on healthy lives and well-being to estimate and project health worker need (see Campbell et al. [16] for earlier estimates). The WHO report defines need as the number of health workers needed to achieve the median level of attainment (25%) for a composite index of 12 tracer health indicators. The decision to define need using the median level of attainment was made by an advisory committee at WHO. The 12 indicators were identified by WHO and the World Bank as proxies of health needs for universal health coverage and the health targets of SDG 3 (Table 1) [17].

**Table 1:** The 12 selected tracer indicators in the SDG composite index threshold and their primary classifications

SDG tracer indicator	Classification
Antenatal care	MNCH
Antiretroviral therapy	ID
Cataract	NCD
Diabetes	NCD
DTP3 immunization	ID
Family planning	MNCH
Hypertension	NCD
Potable water	ID
Sanitation	ID
Skilled birth attendance	MNCH
Tobacco smoking	NCD
Tuberculosis	ID

Source: [1]

Abbreviations: *MNCH* Maternal, Newborn, Child Health, *ID* Infectious Disease, *NCD* Non-communicable Disease

Estimating need proceeded as follows (see the Appendix of the WHO report for details). First, a country was assigned a score from 0 to 12. Countries received 1 point for each indicator where they attained coverage of greater than 80% of the population, such as cataract surgery coverage of over 80% and over 80% of the population not smoking. Next, each indicator was weighted by the global burden of disease that it addresses. Thus, prevention of tobacco smoking addresses an over 12-fold greater disease burden worldwide than would DTP3 vaccination. So, a country can increase its composite index score faster by achieving high tobacco smoking coverage (i.e. a high percentage of the population not smoking) than achieving high DTP3 vaccination coverage. The analytical weights assigned to the 12 indicators were then scaled to sum to 1. Hence, the original 0-12 score becomes a score from 0 to 1 (hereafter the SDG composite score). Then the SDG composite score was regressed on the logarithm of the supply of health workers defined as physicians, nurses, and midwives. The new benchmark was set at the median score of the countries included in the analysis which was 0.25. Regression equations used to estimate need, demand, and supply are presented in the Appendix.

### Demand

The demand for health workers builds on the work of Scheffler and colleagues [18, 19] where countries demand for health workers is correlated with its gross national product (GNP) in the current year and previous year. National income is known as the major predictor of health care spending and hence the demand for health workers. The World Bank paper adds other measures that drive health care demand and the resulting demand for health workers which include the size of the population aged 65 or over [20] and private per capita household out-of-pocket (OOP) spending on medical care which is used as a proxy for the social protection from health care spending [21]. Less generous health coverage leaves individuals to pay more OOP, which is expected to lower the demand for and use of health services. Thus, they expect higher OOP health spending to be negatively correlated with demand for health workers.

In sum, the economic model specifies physician density (dependent variable) as a function of GDP, OOP, and size of the population over 65 years. The model included country fixed effects to account for time-invariant unobservable heterogeneity across countries (i.e. differences in baseline characteristics) that cannot otherwise be controlled (see Liu et al. [2] for more details). The Appendix of this paper details the strengths and weaknesses of this demand model, as well as the supply model discussed in the following section.

### Supply

The WHO report used historical data to project health worker densities (per thousand population) to 2030. Current growth rates were assumed to continue. In a few countries where the rates were implausible (due to the quality of underlying data), linear growth curves for the region-income group were applied to substitute for the country-specific one. This approach is static. It assumed migration patterns, entry in the health profession, and retirement as well as deaths of health workers will remain the same to 2030 (see Scheffler and Cometto et al. [1] for more details).^1^

## Results

**Fig. 1:**
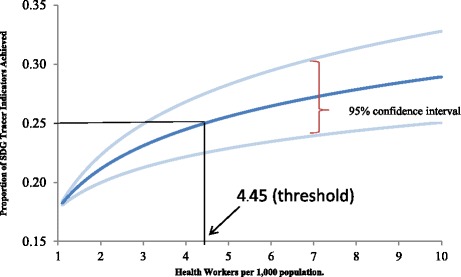
Results from the SDG index composite needs-based method. Source: [1]. Note: Proportion of 12 selected SDG tracer indicators achieved as a function of health workers per 1000 population (*n* = 210 countries and territories). The dark blue curve shows the regression coefficient of health workers; light blue curves show the upper and lower 95% confidence interval of health worker density. Skilled health workers are defined as physicians and nurses/midwives. The SDG tracer indicators were weighted by the global burden of disease each tracer intends to address. The resulting target number is 4.45 workers that achieve the median score (25%) of SDG tracer indicator attainment for all countries analyzed

Here, we summarize the results of the SDG needs-based approach and the demand-based approach. First, we show the relationship between skilled health worker density and the selected SDG tracer conditions (Fig. 1). The vertical axis shows the percent of all the SDG tracer conditions where coverage is achieved, and on the horizontal the number of skilled health workers per thousand population. The new threshold is a density of 4.45 doctors, nurses, and midwives per thousand population, which is set at the median (25%) of attainment of 80% coverage for the 12 selected SDG tracer indicators. We also show the 95% confidence interval of the estimated need line.

We now turn to the needs-based and demand-based estimates. Table 2 shows the estimated need for health workers using the new threshold of 4.45 health workers per thousand in 2013 and the projected need in 2030. The numbers presented in the table differ slightly from the numbers presented in the WHO report as the number of countries was reduced to 165 from 210 to be able to make comparisons across the two papers. The new threshold based on the SDG analysis estimates a global need of over 44.7 million in 2013 which is projected to increase to almost 53 million by 2030, an increase of 18% (for specific details on how the projections were done see Scheffler and Cometto et al. [1]). The largest needs-based estimate for health workers is 17.6 million in lower-middle-income countries in 2013, which is projected to increase to almost 22 million by 2030. However, the largest projected increase in need for health workers is 45% in low-income countries. By region, South-East Asia has the largest needs-based estimate of health workers at 12.4 million, which is projected to increase to 14.7 million by 2030. In contrast, Africa has the largest projected percent increase of need for health workers of 51%.

**Table 2:** Estimated and projected global need of health workers, by World Bank income group and WHO region, 2013 and 2030

	Health worker need		
	2013	2030	% Change
World Bank Income Group (# of countries)			
Low (29)	4 861 904	7 049 048	45%
Lower-middle (44)	17 605 293	21 940 256	25%
Upper-middle (46)	14 617 189	15 934 777	9%
High (46)	7 644 247	8 058 211	5%
WHO Region (# of countries)			
Africa (43)	5 891 071	8 910 473	51%
Americas (28)	5 439 623	6 246 463	15%
Eastern Mediterranean (15)	3 797 769	5 055 625	33%
Europe (50)	5 628 533	5 786 268	3%
South-East Asia (8)	12 433 083	14 712 987	18%
Western Pacific (21)	11 538 553	12 270 476	6%
World (165)	44 728 633	52 982 292	18%

Source: [1]

Note: The World need numbers reported here are roughly 4% below the World need numbers reported in the above reference. The above reference computes need across 210 counties, as opposed to the 165 countries analyzed for this table. Health worker refers to physicians, nurses/midwives, and other health workers

In contrast, the demand for health workers is 48.3 million in 2013 and is projected to increase to 80.3 million by 2030, an increase of 66% (Table 3). The largest demand for health workers is estimated to come from upper-middle-income countries—19 million in 2013 and projected to increase to about 33.3 million by 2030. The smallest demand comes from low-income countries and the Africa region.

**Table 3:** Estimated and projected global demand of health workers, by World Bank income group and WHO region, 2013 and 2030

	Health worker demand		
	2013	2030	% Change
World Bank Income Group (# of countries)			
Low (29)	637 584	1 400 074	120%
Lower-middle (44)	10 897 535	21 682 581	99%
Upper-middle (46)	19 040 552	33 291 730	75%
High (46)	17 690 584	23 780 953	34%
WHO Region (# of countries)			
Africa (43)	1 106 183	2 404 807	117%
Americas (28)	8 826 933	15 288 610	73%
Eastern Mediterranean (15)	3 057 524	6 201 515	103%
Europe (50)	14 178 009	18 158 772	28%
South-East Asia (8)	5 964 318	12 206 786	105%
Western Pacific (21)	15 133 290	25 894 849	71%
World (165)	48 266 256	80 155 338	66%

Source: [2]

Note: Health worker refers to physicians, nurses/midwives, and other health workers

The WHO estimates (for the 165 countries for which demand data exist) the supply of health workers at 41.7 million in 2013, which is projected to be 64.7 million by 2030—an increase of 55% (Table 4). It is no surprise that high-income countries in Europe have the largest supply of health workers but the lowest percent expected increase for the supply of health workers between 2013 and 2030.

**Table 4:** Estimated and projected global supply of health workers, by World Bank income group and WHO region, 2013 and 2030

	Health worker supply		
	2013	2030	% Change
World Bank Income Group (# of countries)			
Low (29)	692 757	1 384 576	100%
Lower-middle (44)	9 867 919	17 958 943	82%
Upper-middle (46)	13 764 139	21 362 033	55%
High (46)	17 385 217	23 948 576	38%
WHO Region (# of countries)			
Africa (43)	1 874 830	3 066 666	64%
Americas (28)	8 385 480	12 742 856	52%
Eastern Mediterranean (15)	2 690 443	4 611 408	71%
Europe (50)	12 692 401	16 803 264	32%
South-East Asia (8)	5 772 250	10 168 591	76%
Western Pacific (21)	10 294 627	17 261 342	68%
World (165)	41 710 032	64 654 127	55%

Source: [1]

Note: The World need numbers reported here are roughly 4% below the World numbers reported in the above reference. The above reference computes need across 210 counties, as opposed to the 165 countries analyzed for this table. Health worker refers to physicians, nurses/midwives, and other health workers

The global needs-based shortage (need minus supply) of health workers using the new SDG threshold of 4.45 health workers per thousand population and demand-based shortage of health workers (demand minus supply) is shown in Table 5. Of most concern is the needs-based shortage in low-income countries or the Africa region of over 4 million health workers which is projected to increase to 6 million by 2030. In contrast, low-income countries show a small surplus (55,000) of health workers vis-a-vis expected demand, which is often due to the lack of financial resources to generate adequate demand to meet population health needs. Finally, the global needs-based shortage was just under 17 million health workers in 2013 and is projected to decrease slightly to 14 million by 2030. The demand-based shortages tell a very different story. The demand-based global shortage for health workers is 6.6 million in 2013 and projected to increase to 15.5 million by 2030—an astounding increase of 136%.

**Table 5:** Estimated and projected global needs-based and demand-based shortages of health workers, by World Bank income group and WHO region, 2013 and 2030 [shortages are positive, surpluses are negative]

	Needs-based shortages (Need-Supply)		Demand-based shortages (Demand-Supply)	
	2013	2030	2013	2030
World Bank Income Group (# of countries)				
Low (29)	4 202 379	5 746 161	− 55 173	15 498
Lower-middle (44)	9 003 163	6 495 262	1 029 616	3 723 638
Upper-middle (46)	3 658 626	1 746 981	5 276 413	11 929 697
High (46)	81 361	74 838	305 367	− 167 623
WHO Region (# of countries)				
Africa (43)	4 194 741	6 088 186	− 768 647	− 661 859
Americas (28)	708 021	503 870	441 453	2 545 754
Eastern Mediterranean (15)	1 569 814	1 508 924	367 081	1 590 107
Europe (50)	78 394	57 749	1 485 608	1 355 508
South-East Asia (8)	6 661 765	4 547 443	192 068	2 038 195
Western Pacific (21)	3 732 794	1 357 071	4 838 663	8 633 507
World (165)	16 945 529	14 063 242	6 556 224	15 501 211

Sources: [1, 2]

Notes: Health worker refers to physicians, nurses/midwives, and other health workers. For demand-based shortages, positive totals represent shortages while negative totals represent surpluses. The total needs-based shortages reported in this table are lower than the totals reported by the WHO report because this table computes needs-based shortages for 165 countries (to correspond with the demand estimates) whereas the WHO report computed needs-based shortages for 210 countries

There is an important distinction in the methods used to calculate global shortages of health workers. The demand-based shortages presented in Table 5 follow the method used by the World Bank paper [2]: shortages and surpluses are added up and a net result is calculated. The WHO report [1] uses a very different method where only countries with shortages are included in the totals. Consistently with the approach of the 2006 WHO report [5], this method does not consider values of health workers in excess of the 4.45 per thousand threshold, a surplus that could potentially offset shortages in countries below the set threshold, because most high- and upper-middle-income countries have a more comprehensive service delivery profile that requires a higher density of health workers. Market forces and the stark wage differentials, moreover, make movements of health workers from higher to lower income settings extremely unlikely.

## Discussion and conclusions

The labor market analysis results presented are global estimates, whose validity depends on the quality of the underlying data and several assumptions and model specifications. Detailed review at the country level is only feasible if accurate and timely workforce data on health workers is systematically and accountably reported as recommended by the Commission. The Commission calls for investment in the analytical capacity of countries to conduct labor market analysis. Improvement in the function of the health labor market will produce a more efficient and effective health system [22]. Given the dire shortage of health workers in low- and middle-income countries, the Commission’s recommendations are critically important.

However, they should be understood as approximations. The data used in both reports are far from ideal. There are missing data for a number of countries and assumptions had to be made at various points in each report. For example, 210 countries were used in the WHO report to estimate need and supply, while 165 countries were used in the World Bank report to estimate demand. For consistency, the need and supply totals presented in this report correspond to the 165 countries included in the World Bank report. A careful read of both reports details the data and analytical limitations.

The empirical results point out some alarming mismatches between need, supply, and demand, and the co-existence of needs-based and demand-based shortages, which pose several health workforce policy dilemmas. The new SDG threshold in 2013 suggests a shortage of just under 17 million health workers with shortages in all but high-income countries. But by 2030, the shortages will be in low-income countries (5.7 million) and lower-middle-income countries (6.5 million). In contrast, the demand shortages are mostly in upper-middle-income countries. The estimated shortages in these countries are 5.3 million in 2013 which is projected to reach 11.9 million by 2030.

The clear result is that upper-middle-income countries and the Western Pacific region will have a very strong economic demand for health workers that will not be met by growth in domestic supply and is therefore likely to exert a strong unmet demand pressure on the supply of health workers from low- and lower-middle-income countries, thereby contributing to greater international labor mobility. These international labor market dynamics will challenge countries who cannot afford to compete financially to retain their health workers. If these challenges are not addressed, there is a strong risk that projected needs-based shortages in low-income countries will be exacerbated. The recommendations of the Global Strategy and further advanced by the Commission are appropriate and essential to address this dilemma. Priority actions include the following.

- In many high-income and upper middle-income countries, the supply of health workers is constrained, raising the cost of health workers and fuelling broader cost escalation in the health sector. In these settings, relaxing, where relevant, over-restrictive barriers to entry into health training and health professions may be required.
- In low-income and some lower-middle-income countries, investments in health worker education should be accompanied by an expansion of the fiscal space to fund positions in the health sector.
- It seems unlikely that low- and low-middle income countries will be able to pay for the health workers they need, even with moderate domestic fiscal space growth by 2030, which means a shared responsibility (shared financing model) between countries to finance all these new jobs will likely be required [23, 24].
- Health workforce strategies should lead to cost-effective resource allocation, deploying interprofessional primary care teams of health workers with a diverse and sustainable skills mix [25], harnessing the potential of community-based and mid-level health workers [26–28].
- Better human resources for health (HRH) evidence is required for effective stewardship of national health labor markets. Of critical importance will be the standardization and interoperability of HRH data, according to the approach recommended through the WHO minimum data set for health workforce registry to establish national health workforce accounts.
- The implementation of the WHO Global Code of Practice on the International Recruitment of Health Personnel and of the WHO Global Strategy on Human Resources for Health: Workforce 2030 should be reinforced and accelerated.
- While these recommendations appear reasonable, the absence of substantial progress on similar such recommendations issued over the last decade calls into question the likelihood of their effective implementation. For example, why would high-income countries address their shortfalls in domestic supply if recruitment of international health workers is cheaper? Changing the status quo is likely to require significant political will and hard-nosed negotiations. There should also be openness to identifying new and innovative approaches to achieving greater labor market equilibria in health that may arise in the broader context of efforts by countries to achieve the SDG objective of universal health coverage [6].

These priorities could be operationalized by a global strategy that involves not only the health ministers of each country, but also ministers of finance. Leadership at the president or prime minister level, especially in low-income countries, is key to success. Creating closely monitored and agreed upon benchmarks which measure the progress of eliminating shortages by 2030 is an important way to ensure that the global workforce strategy is implemented.

## Figures and Tables

**Fig. 2:**
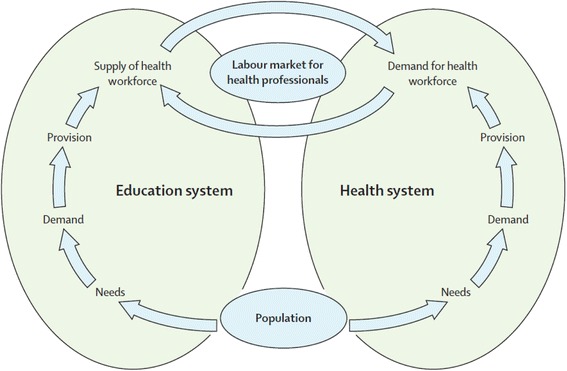
How the labor market for health professionals relates to a country’s education and health care system. Source: [15]

**Fig. 3:**
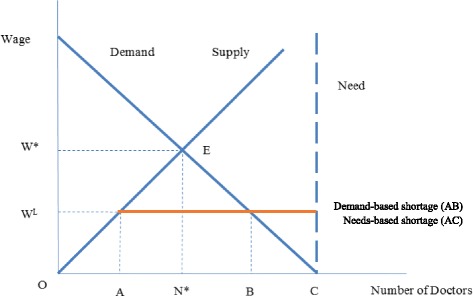
Demand and supply of doctors

## Appendix

### A graphical depiction of need, demand, and supply

Figure 2 shows the demand and supply of doctors though this could apply to nurses or other health workers as well. Demand is related to the spending on health by the government, private insurance, and out-of-pocket payments. The supply of health workers is a function of the training capacity in a country, net migration, deaths, and retirements. In a perfectly functioning labor market, the market clears when the supply of health workers equals the demand at point E. When this happens, the wage would be W* and N* doctors would be employed. However, countries, particularly in resource-constrained low- and middle-income settings, often face binding constraints on the amount of financing that is available for paying health workers as well as rigidities such as government salaries that are set by law across civil servant categories and often do not fully relate to the value of a health worker’s productivity. Thus, the wage offered will be lower, W^L^ rather than W*, resulting in a shortage of doctors represented by length AB. The supply of doctors willing to work at W^L^ is A while the demand for doctors at this wage is B. If the lack of government resources and/or policy prevent wages from rising to W*, the shortage will persist.

The need for doctors is shown as the vertical line which passes through point C though it could be further to the right of C—which is the case in very low-income countries. At a wage W^L^, the number of doctors employed will be A but the need is C, so the needs-based shortage is length AC. In this case, the needs-based shortage is larger than the demand-based shortage by BC (AC minus AB). For high-income countries and upper-middle-income countries, the situation is often reversed. The need line moves to the left inside the demand curve, and the demand-based shortage is often larger than the needs-based shortage (Fig. 3).

### Regression equations used to estimate need, demand, and supply

#### Need

1 $$ \mathrm{SDG}\ \mathrm{composite}\ {\mathrm{score}}_i={\beta}_0+{\beta_1}^{\ast}\mathit{\ln}\left(\mathrm{health}\  \mathrm{workers}\ \mathrm{per}\ 1000\ {\mathrm{population}}_i\right)+{\xi}_i $$

where *ξ*_*i*_ is the disturbance term, and *β*_0_ and *β*_1_ are unknown parameters to be estimated from the model.

#### Demand

2 $$ {\displaystyle \begin{array}{l}\mathit{\ln}\left(\mathrm{physicians}\ \mathrm{per}\ 1000\ {\mathrm{population}}_{it}\right)={\beta}_0+{\beta_1}^{\ast}\mathit{\ln}\left(\mathrm{GDP}\ \mathrm{per}\ {\mathrm{capita}}_{it-1}\right)+\\ {}{\beta_2}^{\ast}\mathit{\ln}\left(\mathrm{GDP}\ \mathrm{per}\ {\mathrm{capita}}_{it-4}\right)+{\beta_3}^{\ast}\mathit{\ln}\left(\mathrm{GDP}\ \mathrm{per}\ {\mathrm{capita}}_{it-5}\right)+{\beta_4}^{\ast}\mathit{\ln}\left({\mathrm{OOPPC}}_{it-2}\right)+\\ {}{\beta_5}^{\ast}\mathit{\ln}\left( Pop{65}_{it-3}\right)+{\mu}_i+{\xi}_{it}\end{array}} $$

where *μ*_*i*_ represents a vector of country fixed effects, *ξ*_*it*_ is the disturbance term, and *β* coefficients are unknown parameters to be estimated from the model. A generalized linear model (GLM) with a normal distribution and identity link function was used to fit a linear regression using a maximum likelihood estimator. Predicted values of logged physician densities from this model were then transformed with an antilog and multiplied by a correction factor ${e}^{\sigma^2/2}$ to account for the skewed distribution.

To avoid endogeneity, GDP per capita, OOP spending per capita (OOPPC), and the size of the population aged 65 or over (Pop65) were all lagged up to 5 years to allow time for such factors to work through the economy and affect the labor market, as other authors have done in previous projection exercises [18, 29]. A stepwise approach was used to select the specific combination of year lags that maximized the predictive power of each variable. Lagged variables that achieved a minimum 1% level of significance after repeated iteration were kept within the model.

#### Supply

WHO separately estimated the growth rate of physician and nurses/midwives density for each country from 1990 to 2013 using the following equations:

3 $$ \mathrm{Physicians}\ \mathrm{per}\ 1000\ {\mathrm{population}}_t={\alpha}_0+{\alpha_1}^{\ast }{\mathrm{year}}_t+{\varepsilon}_t $$

4 $$ \mathrm{Nurses}/\mathrm{midwives}\ \mathrm{per}\ 1000\ {\mathrm{population}}_t={\beta}_0+{\beta_1}^{\ast }{\mathrm{year}}_t+{\varepsilon}_t $$

where *ε*_*t*_ is the random disturbance term and *α*_0_, *β*_0_, *α*_1_, and *β*_1_ are unknown parameters, with the last two parameters representing the linear growth rates to be estimated from the model.

### Strengths and weaknesses of the demand and supply model

Demand model:

- Strengths:
  - Builds on previous models of health worker demand (see [18]) and adds in two factors not previously accounted for but that can have a large effect on demand: out-of-pocket expenditures (i.e., as an indicator for the generosity of health insurance, and thus social protection against catastrophic healthcare spending) and the size of the population aged 65 or over (as an indicator for population aging, which is likely to drive greater demand for care).
  - Inclusion of country fixed effects to account for time-invariant unobservable heterogeneity across countries (i.e., differences in baseline characteristics between countries).
  - Sensitivity analyses showed that the final specification yielded the best predicted values in terms of having the lowest mean error compared to alternative specifications using differently calculated input variables (i.e., percentage of the population aged 65+, out-of-pocket expenditures as a percentage of total per capita health expenditures).
  - Predicted values were also relatively stable using alternative estimates of future values of GDP per capita and size of the population aged 65 or over.
- Weaknesses
  - Cannot take other factors into account that also affect demand, such as changing epidemiology of disease (e.g., epidemiological transition from infectious diseases to non-communicable diseases in many lower-income countries), increased productivity (through technological advance, which can also affect skills mix), changes in the organization of health care delivery
  - Only had sufficient data for physicians to do predictions, had to make global assumptions about nurses/midwives and other types of health care workers. Can build these other cadres in as more data become available.

Supply model:

- Strengths:
  - Maximizes use of historical data for each country to predict future trends
  - Assumes a conservative linear relationship in yearly growth rather than a more aggressive exponential relationship.
- Weaknesses
  - Assumes no change in either the entry or exit of workers into the market, which may be unrealistic (as reviewer mentions example about the aging workforce). Linear trend assumes rate of aging will stay the same when it actually may be increasing, resulting in an overestimate of future supply.
  - Other factors, such as changes in certification/licensing rules, migration, and education capacity could also affect the supply. In many countries, these policy and programmatic changes are enacted to augment the production of health workers, some of which may actually become employed in service delivery. Ignoring these factors would likely result in underestimating future supply.
  - In the end, it is not known what the net effect of these various factors might be on supply without more detailed data on graduation rates, in/out migration of health workers across countries, and retirement to include in the model.

## Abbreviations

DTP3: Diphtheria-tetanus-pertussis
GNP: Gross national product
HRH: Human resources for health
ID: Infectious disease
MNCH: Maternal, newborn, child health
NCD: Non-communicable disease
OOP: Out-of-pocket
SDGs: Sustainable Development Goals
WHA: World Health Assembly
WHO: World Health Organization

## Footnotes

1 This model was used to make a basic prediction of the supply of health workers in the future. The aging of the population in many developed countries will lead to higher retirement rates in the future, implying that the supply for developed countries is likely to be lower than estimated.
